# Erythropoietin Non-hematopoietic Tissue Response and Regulation of Metabolism During Diet Induced Obesity

**DOI:** 10.3389/fphar.2021.725734

**Published:** 2021-09-15

**Authors:** Soumyadeep Dey, Jeeyoung Lee, Constance T. Noguchi

**Affiliations:** Molecular Medicine Branch, National Institute of Diabetes and Digestive and Kidney Diseases, National Institutes of Health, Bethesda, MD, United States

**Keywords:** erythropoietin, erythropoietin receptor, gender-specific, obesity, inflammation, hypothalamus, microglial

## Abstract

Erythropoietin (EPO) receptor (EPOR) determines EPO response. High level EPOR on erythroid progenitor cells gives rise to EPO regulated production of red blood cells. Animal models provide evidence for EPO activity in non-hematopoietic tissue mediated by EPOR expression. Beyond erythropoiesis, EPO activity includes neuroprotection in brain ischemia and trauma, endothelial nitric oxide production and cardioprotection, skeletal muscle wound healing, and context dependent bone remodeling affecting bone repair or bone loss. This review highlights examples of EPO protective activity in select non-hematopoietic tissue with emphasis on metabolic response mediated by EPOR expression in fat and brain and sex-specific regulation of fat mass and inflammation associated with diet induced obesity. Endogenous EPO maintains glucose and insulin tolerance and protects against fat mass accumulation and inflammation. Accompanying the increase in erythropoiesis with EPO treatment is improved glucose tolerance and insulin response. During high fat diet feeding, EPO also decreases fat mass accumulation in male mice. The increased white adipose tissue inflammation and macrophage infiltration associated with diet induced obesity are also reduced with EPO treatment with a shift toward an anti-inflammatory state and decreased inflammatory cytokine production. In female mice the protective effect of estrogen against obesity supersedes EPO regulation of fat mass and inflammation, and requires estrogen receptor alpha activity. In brain, EPOR expression in the hypothalamus localizes to proopiomelanocortin neurons in the arcuate nucleus that promotes a lean phenotype. EPO stimulation of proopiomelanocortin neurons increases STAT3 signaling and production of proopiomelanocortin. Cerebral EPO contributes to metabolic response, and elevated brain EPO reduces fat mass and hypothalamus inflammation during diet induced obesity in male mice without affecting EPO stimulated erythropoiesis. Ovariectomy abrogates the sex-specific metabolic response of brain EPO. The sex-dimorphic EPO metabolic response associated with fat mass accumulation and inflammation during diet induced obesity provide evidence for crosstalk between estrogen and EPO in their anti-obesity potential in female mice mediated in part via tissue specific response in brain and white adipose tissue. Endogenous and exogenous EPO response in non-hematopoietic tissue demonstrated in animal models suggests additional activity by which EPO treatment may affect human health beyond increased erythropoiesis.

## Introduction

Erythropoietin (EPO) is a glycoprotein produced in the adult kidney in a hypoxia responsive manner and functions primarily to regulate erythropoiesis in the bone marrow (Semenza, 2009; Bunn, 2013; Pugh and Ratcliffe, 2017). EPO acts by binding to its cell surface EPO receptor (EPOR) that is expressed at the highest levels on erythroid progenitor cells resulting from transactivation by EPO induced erythroid transcription factors, GATA1 and TAL1 (Broudy et al., 1991; Zon et al., 1991; Kassouf et al., 2010; Rogers et al., 2012). EPO stimulation of erythroid progenitor cells activates JAK2/STAT and other signaling pathways to promote cell survival, proliferation and differentiation, resulting in the production of 200 billion new red blood cells daily in the human body (Witthuhn et al., 1993; Kuhrt and Wojchowski, 2015; Bhoopalan et al., 2020). Following the cloning of the human *EPO* gene (Lin et al., 1985; Jacobs et al., 1985), recombinant human *EPO* was approved for clinical use by the United States Food and Drug Administration in 1989 primarily for treatment of anemia associated with chronic renal disease. For three decades, EPO treatment in hemodialysis patients has reduced exposure to blood transfusions and associated with improved physical performance (Paoletti and Cannella, 2006; Jelkmann, 2013; Wright et al., 2015).

Targeted deletion of *EPO* (*EPO−/−*) or *EPOR* (*EPOR−/−*) in mice results in embryonic death due to severe anemia (Wu et al., 1995; Lin et al., 1996). Although EPOR expression in erythroid tissue is required for life (Suzuki et al., 2002), EPOR expression is not restricted to erythroid tissue. EPO response in animal models provides evidence for EPOR mediated EPO activity in non-hematopoietic tissues that include EPO response to ischemia and injury in brain, heart and skeletal muscle, and EPO stimulated bone remodeling (Suresh et al., 2020a). This review focuses on the metabolic activity of EPO such as regulation of fat mass and inflammation during diet induced obesity and sex-dimorphic EPO response in fat and brain (Table 1).

**TABLE 1:** Erythropoietin regulation of fat mass and inflammation in mice during diet induced obesity.

Mouse model	EPO status	Physiologic response	Physiologic response	References
WT (C57BL/6)	EPO gene electrotransfer in skeletal muscle	Fat mass	-EPO in females on high fat diet (HFD) decreased body weight/fat mass and improved glucose tolerance	Hojman et al. (2009)
	EPO treatment	Fat mass	-EPO decreased blood glucose (also WT BALB/c)	Katz et al. (2010)
	EPO treatment	Fat mass	-In males on HFD, EPO ≥ 150 U/kg reduced body weight/fat mass, ≥ 300 improved glucose tolerance, at 1000 U/kg increased physical activity	Foskett et al. (2011)
	EPO treatment	Fat mass	-EPO in males reduced body weight/fat mass accumulation on HFD and reduced body weight on NCD	Teng et al. (2011)
	EPO treatment	Fat mass	-EPO Increased WAT metabolic activity, mitochondria content, oxygen consumption, brown fat program in males	Wang et al. (2013)
	EPO treatment	WAT inflammation	-EPO reduced obese WAT inflammation, inflammatory cytokine production, macrophage infiltration, shifted WAT macrophages to anti-inflammatory phenotype in males	Alnaeeli et al. (2014)
	EPO treatment	Fat mass	-EPO reduced body weight, fat mass and activated brown adipose tissue during HFD.	Kodo et al. (2017)
	EPO treatment	Fat mass	-EPO reduced body weight/fat mass accumulation during NCD and HFD in males.	Zhang et al. (2017)
			-EPO did not change body weight/fat mass in females	
			-EPO reduced body weight/fat mass accumulation in ovariectomized females on HFD.	
	EPO treatment	Bone marrow adipose tissue	-EPO reduced bone marrow adipocytes and bone without change in WAT.	Suresh et al. (2019)
	EPO treatment	Bone marrow adipose tissue	-EPO reduced HFD increase in bone marrow adipocytes independent of change in whole fat mass in males and females	Suresh et al. (2020c)
	Brain EPO administration	Fat mass Hypothalamus inflammation	-EPO reduced body weight/fat mass accumulation and hypothalamus inflammation during HFD in males	Dey et al. (2020)
			-EPO did not change body weight/fat mass accumulation and hypothalamus inflammation during HFD in females	
			-EPO reduced body weight/fat mass accumulation and hypothalamus inflammation during HFD in ovariectomized females	
	Brain EPO administration	Fat mass	-EPO decreased body weight/fat mass, increase lean mass, reduced food intake in males	Wang et al. (2020)
tg6 (C57BL/6)	High transgenic human EPO	Fat mass	-Males and females have lower body mass	Katz et al. (2010)
		Bone marrow adipose tissue	-Males and females have reduced bone marrow adipocytes and bone	Suresh et al. (2019)
tg21 (C57BL/6)	High brain transgenic human EPO	Fat mass Hypothalamus inflammation	-Males have reduced body weight/fat mass accumulation and hypothalamus inflammation during HFD	Dey et al. (2020)
			-No change in body weight/fat mass accumulation and hypothalamus inflammation during HFD in females	
			-Ovariectomized females have reduced body weight/fat mass accumulation and hypothalamus inflammation during HFD	
*ΔEPOR_E_* (C57BL/6)	EPOR restricted to erythroid tissue	Fat mass	-Males and females have increased fat mass, glucose intolerance and insulin resistance.	Teng et al. (2011)
		Bone marrow adipose tissue	-Males and females have reduced trabecular bone and increase bone marrow adipocytes that decreases with EPO treatment.	Suresh et al. (2019)
			-EPO reduced bone marrow adipocytes without change in WAT	
		WAT inflammation	-Increased WAT inflammation in obese *ΔEpoR_E_* mice.	Alnaeeli et al. (2014)
*EPOR^(aP2KO)^* (C57BL/6)	*EPOR^loxP/loxP^*;aP2-Cre fat knockout	Fat mass	-Males have increased susceptibility to diet induced obesity	Wang et al. (2013)
*EPOR^(aP2KO)^* (129J-C57BL/6-FVB/N)	*EPOR^loxP/loxP^*;aP2-Cre fat knockout	Body weight	-Body weight unchanged on NCD or HFD	Luk et al. (2013)
		WAT Inflammation	-WAT inflammation unchanged	
*EPOR^(nestinKO)^* (C57BL/6)	*EPOR^loxP/loxP^*;nestin-Cre neural knockout	Fat mass	-Increased inflammation and weight gain during diet induced obesity in males and not females	Dey et al. (2020)
		Hypothalamus inflammation		
*Ob/ob* (C57BL/6)	Obesity model	Body weight	-In males, EPO reduced body weight	Katz et al. (2010)
		Fat mass	-EPO reduced body weight/fat mass accumulation	Teng et al. (2011)
*PTP1B−/−* (BALB/c)	Obesity model	Body weight	-In males, EPO reduced body weight gain, Hb A1c	Katz et al. (2010)
*STAT6−/−* (C57BL/6)	Defective anti-inflammatory like macrophages	WAT inflammation	-In obese males, EPO does not shift WAT macrophages to anti-inflammatory phenotype observed in obese WT	Alnaeeli et al. (2014)
IL4−/− (C57BL/6)				
*ERα−/−* (C57BL/6)	Estrogen receptor α knockout	Fat mass	-EPO reduced HFD body weight/fat mass accumulation in WT males, in *ERα−/−* males and females	Lee et al. (2021)
*ERα^(adipoKO)^* (C57BL/6)	*ERα^loxP/loxP^*;adiponectin-Cre fat knockout	Fat mass	-EPO reduced HFD body weight/fat in *ERα^(adipoKO)^* females	Lee et al. (2021)
		WAT inflammation	-EPO reduced obese WAT inflammation in *ERα^(adipoKO)^* females but not in WT females	

## Erythropoietin Activity in Erythroid Development and Non-hematopoietic Tissue

EPO regulates survival, proliferation, and differentiation of erythroid progenitor/precursor cells. Erythropoiesis in mammals begins with primitive erythropoiesis in blood islands in the extraembryonic yolk sac at E7.5 in mice and erythroid cells transiently circulate as large, nucleated cells that ultimately enucleate (Palis et al., 1999). Primitive erythroid progenitors exhibit an aerobic glycolytic gene expression profile characteristic of cancer and rapidly proliferating cells and form larger sized colonies in culture at low oxygen (Isern et al., 2011). EPO signaling is not required for formation of primitive erythropoiesis but is required for appropriate rate of terminal maturation of primitive erythroid precursors (Malik et al., 2013). EPO support for maturation of primitive erythroid precursors may be produced in the yolk sac and/or neuroepithelial cells (Yasuda et al., 2002; Suzuki et al., 2013). EPO produced in the fetal liver and adult kidney promotes definitive erythropoiesis in the fetal liver and after birth in the bone marrow giving rise to circulating, enucleated mature red blood cells (Koury et al., 1988). During definitive erythropoiesis, EPO signaling is required for erythroid progenitor/precursor cell survival, proliferation and differentiation beyond the colony forming unit-erythroid (CFU-E) stage. *EPO−/−* and *EPOR−/−* mice die *in utero* at E13.5 due to lack of definitive erythropoiesis and the resultant severe anemia (Wu et al., 1995; Lin et al., 1996).

**FIGURE 1:**
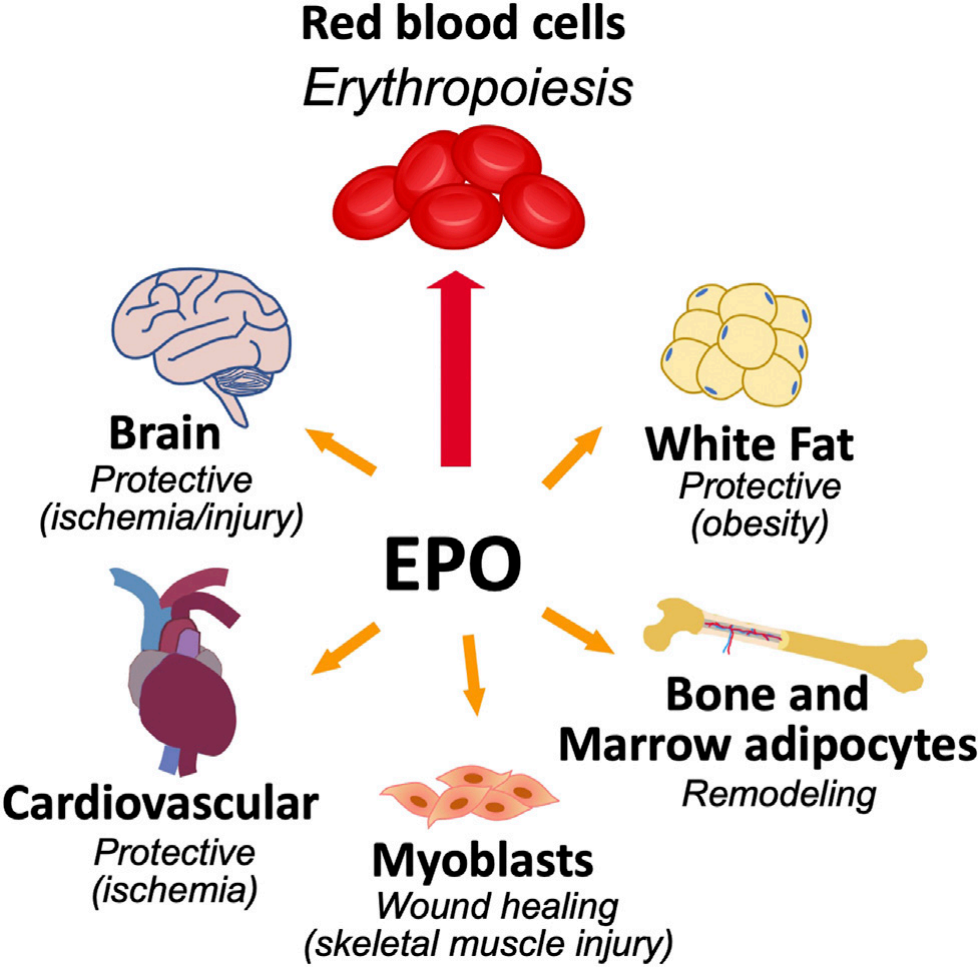
Erythropoietin activity in erythroid and select non-hematopoietic tissues. The primary function of EPO is to regulate the production of red blood cells from erythroid progenitor cells that express high level of EPOR. EPOR expression in non-hematopoietic tissue provides for EPO response that includes brain for neuroprotection and metabolic response, cardiovascular system for cardioprotection and endothelial regulation of vascular tone and oxygen delivery, skeletal muscle maintenance and wound healing, bone remodeling, and metabolic response in white adipose tissue including sex-dimorphic regulation of fat mass and inflammation associated with diet induced obesity.

While EPO activity was initially thought to be limited to regulation of erythropoiesis, animal studies indicate activity of endogenous EPO and exogenous EPO in non-erythroid tissues such as neurons and brain (Masuda et al., 1993; Sakanaka et al., 1998; Yu et al., 2002; Tsai et al., 2006), vascular endothelium and heart (Anagnostou et al., 1990; Kertesz et al., 2004; Cai and Semenza, 2004), myoblasts and skeletal muscle (Ogilvie et al., 2000; Jia et al., 2012), fat (Teng et al., 2011; Alnaeeli et al., 2014; Zhang et al., 2017), and bone (Figure 1) (Holstein et al., 2007; Hiram-Bab et al., 2015; Suresh et al., 2019). During development, EPOR is expressed in non-hematopoietic tissue such as heart and brain and hypoplasia in embryonic heart and brain of *EPOR−/−* mice add further support for direct non-hematopoietic tissue response to EPO (Kertesz et al., 2004; Yu et al., 2001; Yu et al., 2002; Suresh et al., 2020a).

## Erythropoietin Regulation of Fat Mass by Erythropoietin Receptor Expression in Nonerythroid Tissue

### Mice With Erythropoietin Receptor Restricted to Erythroid Cells

**FIGURE 2:**
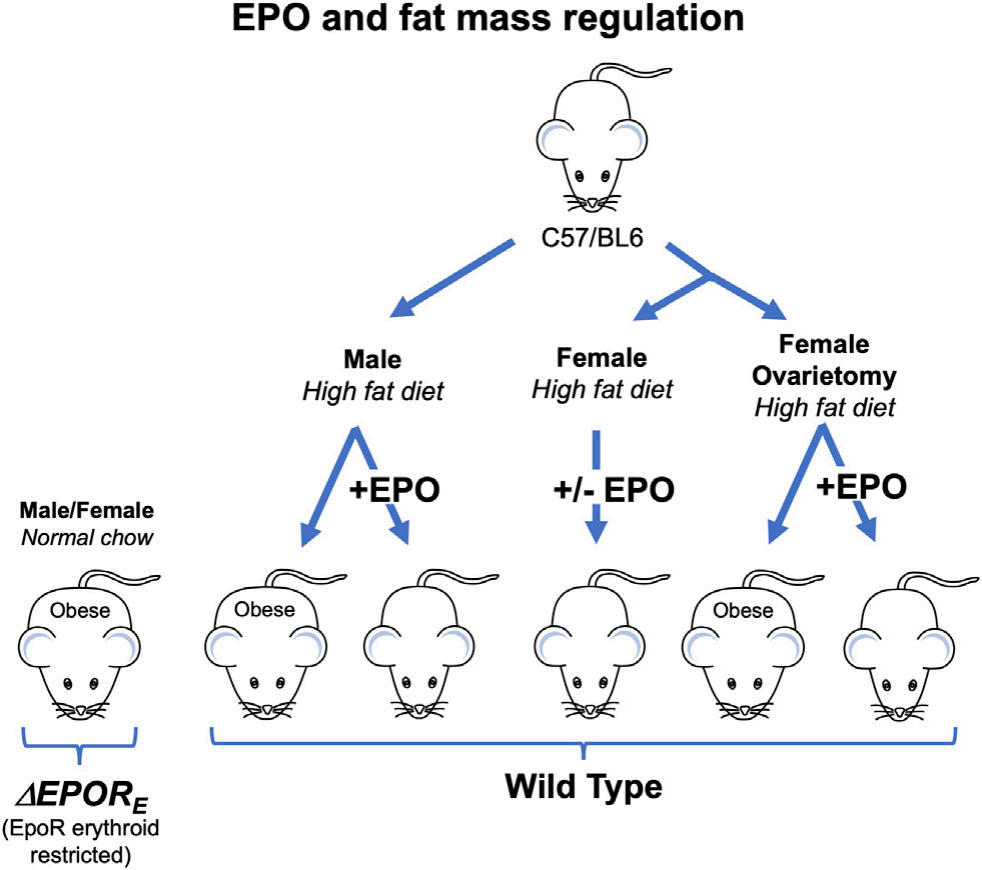
Erythropoietin regulation of fat mass in mice is sex-specific. Male and female *ΔEPOR*_*E*_ mice with EPOR restricted to erythroid tissue exhibit increased gain in body weight and fat mass on normal chow diet and become obese. Male, but not female, wild type (C57BL/6) mice fed high fat diet become obese while male mice on high fat diet and treated with EPO show increase hematocrit without change in fat mass. Ovariectomy eliminates the protective effect of estrogen against diet induced obesity in females and ovariectomized mice fed high fat diet become obese, but not ovariectomized mice treated with EPO.

Endogenous EPO is involved in the regulation of fat mass (Teng et al., 2011), and EPOR is highly expressed in white adipose tissue (WAT) including adipocytes and stromal vascular fraction (SVF) in WT mice (Alnaeeli and Noguchi, 2015). A transgene with EPOR expression restricted to erythroid tissue is able to rescue *EPOR−/−* mice from severe embryonic anemia and gives rise to viable *ΔEPOR*_*E*_ mice with EPOR restricted to erythroid tissue (Suzuki et al., 2002). *ΔEPOR*_*E*_ mice develop obesity and glucose intolerance but exhibit normal erythropoiesis (Figure 2; Table 1) (Teng et al., 2011). In WT mice, EPO treatment reduces fat mass and body weight in males fed normal chow and decreased fat mass gain and body weight accumulation in males on high fat diet (Figure 2) (Teng et al., 2011; Zhang et al., 2017). This provides evidence that exogeneous EPO reduces fat mass accumulation in males. EPO regulation of fat mass could be related to inhibition of preadipocyte differentiation by EPO-stimulated PPARγ phosphorylation (Teng et al., 2011). Brown adipose tissue activation has also been suggested to mediate EPO stimulated increase in oxygen consumption, improved glucose tolerance and reduction in body weight and fat mass in young, male mice on high fat diet treated (Kodo et al., 2017). EPO was also associated with improved diabetes-associated cognitive dysfunction in male rodents (Wang et al., 2017; Othman et al., 2018). EPO administration in male WT mice increased energy expenditure and total activity and reduced food intake in WT while loss of endogenous EPO activity in non-hematopoietic tissue in *ΔEPOR*_*E*_ mice led to decreased energy expenditure with decreased total activity compared with WT mice (Teng et al., 2011).

### Mice With Deletion of Erythropoietin Receptor in Adipose Tissue

The EPO induced metabolic activity is related to promotion of brown fat like feature in white adipose tissue, which is mediated by PPARα coordinating with Sirt1 (Wang et al., 2013). The direct adipocyte EPO response was determined using mice with adipocyte-specific deletion of EPO receptor (*EPOR*_*E*_^*(aP2KO)*^). *EPOR*_*E*_^*(aP2KO)*^ male mice on a C57BL/6 background exhibited obesity and glucose intolerance during high fat diet feeding (Table 1) (Wang et al., 2013). *EPOR*_*E*_^*(aP2KO)*^ mice showed decreased AKT phosphorylation in white adipose tissue while EPO treatment increased AKT phosphorylation in WT mice, suggesting EPO activity regulates AKT activation, which may contribute to glucose and energy homeostasis via insulin signaling (Jordan et al., 2011; Wang et al., 2013). In addition to EPOR expression in white adipose tissue mediating direct metabolic EPO activity, background strain also appears to affect metabolic response of *EPOR*_*E*_^*(aP2KO)*^ mice (Luk et al., 2013).

### Erythropoietin Regulation of Fat Mass Accumulation is Sex Specific

Compared with males, female *ΔEPOR*_*E*_ mice become obese earlier with a greater proportionate accumulation of body fat mass suggesting sex specific metabolic effects of EPO in mice with the loss of EPOR in nonerythroid tissues (Teng et al., 2011). Increased circulating EPO by EPO administration in male mice or by over-expression in skeletal muscle via gene electrotransfer in female mice reduced body weight and fat mass during high fat diet induced obesity (Hojman et al., 2009; Foskett et al., 2011). In mouse models of diabetes and obesity, EPO treatment in male mice with protein Tyr phosphatase knock out (*PTP1B−/−*) and in *ob/ob* mice decreased blood glucose and body weight gain (Teng et al., 2011; Katz et al., 2010). While EPO treatment decreased fat mass accumulation in WT male mice and stimulated expression of mitochondrial oxidative genes in white adipose tissue, this was not observed in WT female mice (Table 1) (Zhang et al., 2017; Katz et al., 2010). Of note, the increased hematocrit levels and improved glucose tolerance by exogeneous EPO were observed in both male and female WT mice (Zhang et al., 2017). The EPO associated sex-dimorphic fat mass regulation can be linked to estrogen production in female mice, which contributes to energy metabolism, insulin sensitivity, and lipid metabolism (Xu and López, 2018). After ovariectomy surgery, mice on high fat diet treated with EPO showed decreased fat mass gain, while estradiol supplementation abrogated this EPO response (Figure 2) (Zhang et al., 2017). Estrogen dependent sex-specific EPO activity has also been demonstrated in mice for EPO induction in the uterine endometrium contributing to angiogenesis during the estrus cycle (Yasuda et al., 1998) and for hypoxia induced carotid body dependent EPO ventilatory response (Soliz et al., 2012).

Estrogen receptor alpha (*ERα*) knockout (*ERα−/−*) mice were shown to have similar phenotypes with menopausal women who have symptoms such as weight gain and reduction in metabolism (Arao et al., 2018). Female *ERα−/−* mice showed similar body weight compared with male WT and male *ERα−/−* mice but the body weights of *ERα−/−* mice were greater compared to female WT at 4 months on normal chow diet (Lee et al., 2021). EPO administration increased hematocrit levels and did not change fat mass in female *ERα^−/−^* and WT mice on normal chow diet, which is consistent with EPO activity in ovariectomized mice on normal chow diet (Zhang et al., 2017; Lee et al., 2021). In contrast, on high fat diet, EPO metabolic regulation of body weight, fat mass, and glucose homeostasis was observed in *ERα−/−* mice and in mice with ERα deleted in adipose tissue (*ERα^(adipoKO)^*) (Table 1) (Lee et al., 2021). The exogenous EPO treatment reduced fat mass in female *ERα−/−* but there was no change in fat mass in female WT mice during high fat diet feeding (Lee et al., 2021). The EPO fat regulation in *ERα−/−* mice is related to browning of white adipocyte, mediated by decreased white fat associated genes, Psat1, Wdnm1-like, Sepina3K and induced brown fat specific uncoupling protein 1 (UCP1) (Lee et al., 2021). EPO stimulated increase in hematocrit was comparable in all mouse groups indicating EPO regulation of fat mass is independent of EPO-induced red blood cell production. These results suggest cross talk between EPO and estrogen and that direct ERα response in adipose tissue to modulates EPO action in metabolic regulation beyond erythropoiesis, especially in female mice.

In humans, hypoxia results in increased EPO level and increased erythropoiesis and iron utilization with elevated blood hemoglobin and hematocrit as individuals ascend to high altitude (Smith et al., 2008; Gassmann and Muckenthaler, 2015). EPO regulation of metabolism and fat mass in mice (Teng et al., 2011; Zhang et al., 2017) may explain in part the association of reduced incidence of obesity in military recruits in the United States (∼94% male) that reside at high altitude (Voss et al., 2014). Full-heritage Pima Indians exhibit a high prevalence of obesity and type 2 diabetes (Smith et al., 1996; Pavkov et al., 2007), and assessment of endogenous plasma EPO level in a subset 79 individuals showed the expected negative association with hemoglobin (*p* = 0.005) (Reinhardt et al., 2016). In addition, males exhibited an inverse association of endogenous EPO level with percent weight change per year (*p* = 0.02) while females exhibited a positive association (*p* = 0.02). This provides evidence for sex specific correlation of endogenous EPO with weight change associated with weight loss in men and weight gain in women and independent of EPO regulation of erythropoiesis (Reinhardt et al., 2016).

### Erythropoietin Promotes Bone Marrow Fat and Bone Remodeling in Male and Female Mice

In mice, EPO stimulated erythropoiesis is accompanied by decrease bone marrow adipocytes independent of changes in fat mass in white adipose tissue and by bone loss (Suresh et al., 2019). EPO activity in bone remodeling is context dependent and in animal models EPO can increase bone healing or promote bone loss during EPO stimulated erythropoiesis (Hiram-Bab et al., 2017; Suresh et al., 2020a). EPO increases bone healing in mouse fracture models (Holstein et al., 2007; Garcia et al., 2011; Shiozawa et al., 2010), and accelerates new bone formation in rats and rabbits (Li et al., 2015; Omlor et al., 2016; Mihmanli et al., 2009). However, trabecular bone loss accompanies EPO stimulated erythropoiesis in mice treated with EPO or expressing high level of transgenic EPO (Suresh et al., 2019; Hiram-Bab et al., 2015; Singbrant et al., 2011). Bone loss associated with EPO treatment is mediated via EPOR expression in osteoblasts and B-cells (Suresh et al., 2019; Suresh et al., 2020b; Deshet-Unger et al., 2020). Mice expressing high transgenic EPO produce increased osteoclast numbers in the femur, and osteoclasts and calvarial osteoblasts exhibit increased differentiation in culture, consistent with the significant reduction in trabecular bone observed *in vivo* (Hiram-Bab et al., 2015; Suresh et al., 2019).

The potential for endogenous EPO to affect bone health in human was observed in a study of bone fracture in elderly Swedish men with normal renal function that revealed high endogenous levels of EPO correlated with increased fracture risk (Kristjansdottir et al., 2019). Although incidence of hip fractures decreased among the general population in the United States after 1990, hip fracture incidence increased among hemodialysis patients coincident with EPO use and dose escalation up to 2004 (Suresh et al., 2021). Average EPO dose per week then decreased relating to reports of adverse cardiovascular events in clinical trials designed to achieve normal hemoglobin levels and the FDA “Black Box” warning against EPO use for hemoglobin targets over 12 g/dl, and to changes in Medicare/Medicaid reimbursement in 2007. Incidence of hip fracture among hemodialysis patients also decreased after 2004. Multivariable analysis of United States Renal Data System (USRDS) datasets for 1997–2013 revealed EPO treatment as a previously unrecognized independent risk factor for hip fracture in hemodialysis patients (Suresh et al., 2021). These findings illustrate the potential of EPO studies in animal models to be informative for EPO use and human health.

### Erythropoietin Receptor Expression in Brain and Brain Erythropoietin Regulates Fat Mass During High Fat Diet Feeding

EPOR in brain detected by a transgenic reporter gene is highly expressed in mice at mid-gestation in the neural tube, and decreases with developmental age to 1–3% of hematopoietic tissue at birth (Liu et al., 1997). EPOR was shown to be expressed in neurons and EPO binding was demonstrated for neural cells contributing to a neuroprotective effect, and in select locations in the mouse brain including the hippocampus, cortex and midbrain areas (Masuda et al., 1993; Morishita et al., 1997; Digicaylioglu et al., 1995).

*EPOR−/−* mice show changes in genes associated with regulation of neural progenitor cell proliferation, maturation, and survival (Sollinger et al., 2017). Human EPO and EPOR expression was detected in the developing central nervous system and in brain as early as 5 weeks post-conception and specific distribution changed with development (Juul et al., 1998; Juul et al., 1999). EPO expressed in human, non-human primate and murine brain by astrocytes and neurons is upregulated by hypoxia providing further support for the biological potential of EPO activity on the brian side of the blood brain barrier (Masuda et al., 1994; Marti et al., 1996). Even prior to severe anemia, embryonic *EPOR−/−* mice show reduced neuroepithelium and neural progenitor cells, increased brain apoptosis and neural cells with increased sensitivity to hypoxia (Liu et al., 1997; Yu et al., 2002). Mice that retain EPOR expression in hematopoietic tissue but lack EPOR expression in neural cells exhibit no gross morphological defects but show reduced neural cell proliferation and viability, sensitivity to glutamate damage and post-stroke neurogenesis (Tsai et al., 2006; Chen et al., 2007). EPO treatment in adult animal models shows increase in newly generated interneurons and protection with EPO preconditioning prior to ischemia or middle cerebral artery occlusion (Sakanaka et al., 1998; Sadamoto et al., 1998; Bernaudin et al., 1999; Shingo et al., 2001). EPO protective activity in brain is suggested to be associated with neurogenesis and revascularization as described for a rodent model of neonatal hypoxia/ischemia (Iwai et al., 2007). Demonstration of EPO for neuroprotection in clinical trials for stroke (Ehrenreich et al., 2002; Ehrenreich et al., 2009; Ehrenreich et al., 2011) or for very preterm infants (Natalucci et al., 2020; Juul et al., 2020a) remains elusive. High dose EPO in very preterm infants reduced the transfusion needs in this population, but at 2 years of age lower risk of severe neurodevelopment impairment was not observed (Juul et al., 2020a; Juul et al., 2020b).

The hypothalamus is a master regulatory site of appetite and energy expenditure via the function of orexigenic agouti-related peptide (AgRP)/neuropeptideY (NPY)-producing neurons and anorexigenic proopiomelanocortin (POMC)-producing neurons. The POMC neurons are localized in the arcuate nucleus region of the hypothalamus. Ablation of POMC neurons and loss of POMC-derived neurotransmitters lead to obesity, underscoring the importance of POMC neurons in regulation of energy homeostasis (Yaswen et al., 1999; Xu et al., 2005). Leptin, a hormone predominantly produced from the white adipose tissue, plays a critical role in hunger suppression by activating the POMC neurons. EPO and leptin are both members of the class-I cytokine superfamily (Ouyang and He, 2003; Frühbeck, 2006) and act through the JAK/STAT signaling pathway (Constantinescu et al., 2001; Mancour et al., 2012). LEPR and EPOR are expressed in the POMC neurons and both leptin and EPO stimulation leads to increase in POMC expression in the hypothalamus. Both leptin and EPO function is mediated via STAT3 activation and results in decreased food intake (Teng et al., 2011; Bates et al., 2003; Dey et al., 2016). Moreover, optimum POMC neuron stimulation by leptin requires the presence of EPOR and *ΔEPOR*_*E*_ mice show lower POMC expression, contributing to the obesity, insulin resistance, and glucose intolerance seen in these mice (Teng et al., 2011).

### High Fat Diet Feeding in Mice With Deletion of Erythropoietin Receptor in Neural Cells

EPO regulation of metabolism through its function in the neuronal cells has been studied in mice by *EPOR* gene knock out in the nestin expressing cells (*EPOR^(nestinKO)^*; Table 1) (Dey et al., 2020). Differences between WT and *EPOR^(nestinKO)^* mice are visible primarily during metabolic stress conditions. *EPOR^(nestinKO)^* mice show greater high fat-diet induced weight gain and glucose intolerance, particularly in male mice. In contrast, females exhibit estrogen protection to diet induced obesity and high fat diet does not increase weight gain and glucose intolerance in female *EPOR^(nestinKO)^* (Dey et al., 2020).

### Regulation of Metabolism by Elevated Brain Erythropoietin is Sex-Dimorphic

In mice, transgenic over-expression of EPO in the brain (tg21) provides protection from metabolic stress resulting from high fat-diet feeding (Table 1) (Dey et al., 2020). Transgenic PDGF B-chain promoter driving expression of human EPO in tg21 mice provides a model to study EPO over-expression in the brain (Kilic et al., 2005). Male and female tg21 mice show improved glucose tolerance on normal chow and high fat diet feeding, and male tg21 mice on high fat-diet feeding show lower weight gain and fat mass gain compared to wild type control mice with normal EPO levels (Dey et al., 2020). In comparison to male mice, female mice are protected from high fat-diet induced weight gain and metabolic stress. Ovariectomized control wild type mice lose the protective estrogen effect against high fat diet induced weight and show increased fat mass accumulation, while ovariectomized tg21 mice can still prevent this metabolic stress due to the elevated EPO levels in the brain (Dey et al., 2020). Administration of recombinant human EPO in the brain via intracerebroventricular osmotic pump or via a cannula implanted into the hypothalamus can also protect the male mice from gaining weight and fat during high fat diet feeding (Dey et al., 2020; Wang et al., 2020).

### Erythropoietin Regulation of Adrenocorticotropic Hormone in the Pituitary Gland

In the pituitary gland, the level of EPOR expression is comparable to that in the hypothalamus, and EPO functions in regulating the secretion of the adrenocorticotropic hormone (ACTH) from the pituitary (Dey et al., 2015). Both endogenous EPO and EPO treatment affect serum ACTH levels (Dey et al., 2015). ACTH is derived from the POMC precursor peptide in the pituitary and EPO has been found to regulate its secretion by controlling the Ca2+ signaling pathways. EPO decreases the intracellular Ca2+ levels, thereby reducing the secretion of ACTH from the pituitary gland. Accordingly, the *ΔEPOR*_*E*_ mice show higher serum ACTH levels even in young mice at 3 weeks of age, that contributes to the development of the metabolic syndrome in these mice (Dey et al., 2015). The hypothalamus and the pituitary gland are part of the hypothalamus-pituitary-adrenal gland (HPA) axis that regulate the stress response pathways. With EPO produced by cells in the brain, such as astrocytes, and with EPO-stimulated POMC expression in the hypothalamus and EPO-inhibited ACTH secretion in the pituitary, EPO signaling contributes to the hypothalamic–pituitary axis as a major regulator of glucose metabolism and energy homeostasis.

## Erythropoietin Regulation of Inflammation During Diet Induced Obesity

### Sex Dimorphic Erythropoietin Regulation of Inflammation in Obese White Adipose Tissue

Obesity-induced insulin resistance increases the risk to develop type 2 diabetes and causes macrophage infiltration in white adipose tissue through chronic inflammation (Guilherme et al., 2008). With ongoing white adipose tissue inflammation, M1 pro-inflammatory macrophage infiltration increases, and the macrophage population in white adipose tissue shifts from anti-inflammatory M2-like cells to predominantly pro-inflammatory M1-like cells. M1 macrophages release the pro-inflammatory cytokines, which interfere with insulin signaling (Lauterbach and Wunderlich, 2017). In addition to white adipocytes, macrophages in the stromal vascular fraction of white adipose tissue express EPOR, especially in obese mice (Alnaeeli et al., 2014). Crown-like structures are known as indicators of proinflammatory process in adipose tissue, which are macrophages mostly derived from monocytes surrounding necrotic adipocytes. Estrogen in female mice is protective against obesity and female mice on high fat diet show a blunted increase in adiposity and inflammatory response compared with male mice (Singer et al., 2015). High fat diet feeding for 16 weeks showed increase crown-like structures only in male mice. Two weeks of EPO treatment in obese mice (10 weeks of high fat diet-feeding) did not affect body weight but dramatically improved inflammation, reduced the number of crown-like structures, and decreased macrophage infiltration in male WT mice (Table 1) (Alnaeeli et al., 2014). These anti-inflammatory effects are attributed to direct EPOR response in macrophage via STAT3 phosphorylation, which is associated with reduced proinflammatory gene expression with increased anti-inflammatory cytokine, IL-10 expression. EPO also stimulated macrophage subtype shift toward the anti-inflammatory M2-like cells, which requires IL-4/STAT6 signaling (Table 1). This suggests that the EPO anti-inflammatory activity in obese white adipose tissue is independent from EPO regulation of body weight and fat mass. Endogenous EPO in white adipose tissue contributes to an anti-inflammatory phenotype in male mice. During high fat diet induced obesity, *∆EPOR*_*E*_ male mice exhibited higher circulating inflammatory monocyte numbers, increased macrophage inflammatory infiltrates, and enhanced crown like structures although their body weight and fat mass were similar to WT males (Alnaeeli et al., 2014).

In obese female WT mice (10 weeks of high fat diet feeding), EPO treatment for 2 weeks did not promote the anti-inflammatory response in white adipose tissue observed in male mice (Lee et al., 2021). However, EPO treatment for 2 weeks in obese female ERα^adipoKO^ decreased pro-inflammatory associated genes, TNFα and iNOS in white adipose tissue, and 4 weeks EPO treatment in female *ERα−/−* mice on high fat diet reduced white adipose tissue TNFα expression (Table 1) (Lee et al., 2021). Thus, these findings suggest that estrogen activity in female mice interferes with the EPO anti-inflammatory activity in white adipose tissue associated with diet induced obesity and that loss of adipocyte specific ERα allows for the anti-inflammatory response by EPO.

### Sex-specific Erythropoietin Regulation of Inflammation in Hypothalamus During High Fat Diet Feeding

**FIGURE 3:**
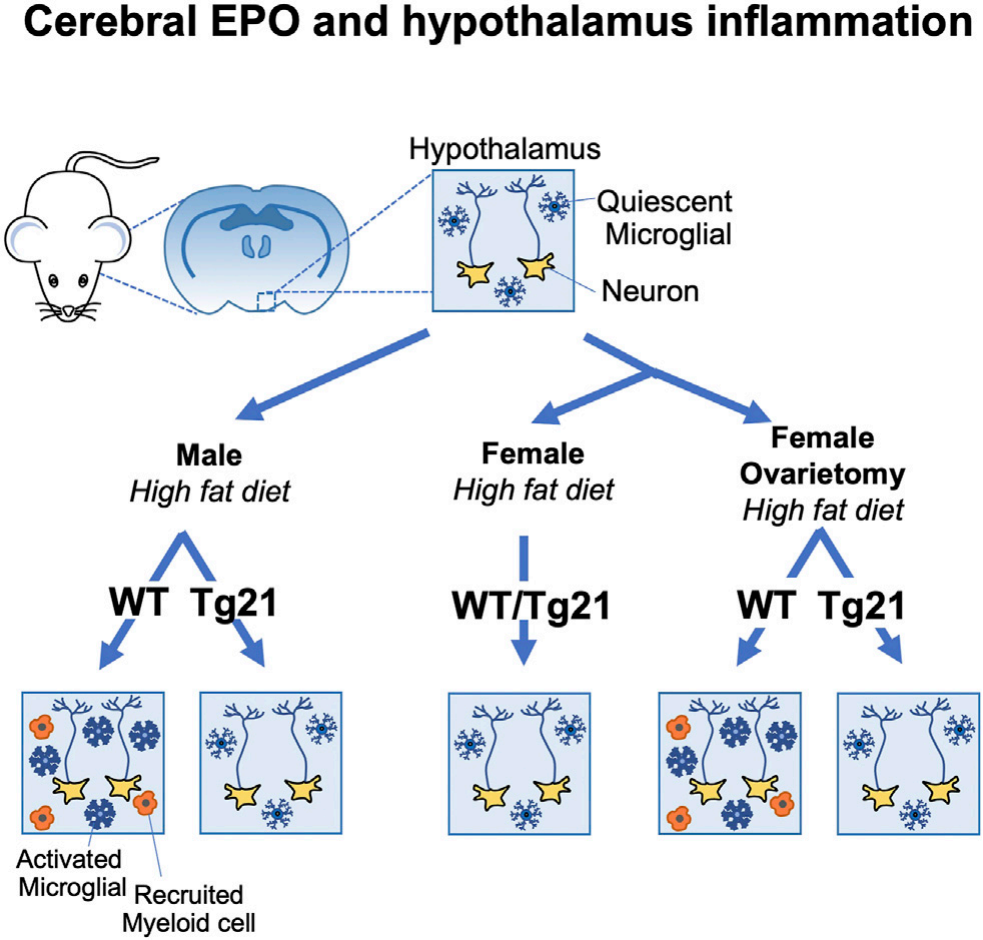
Brain erythropoietin reduction of hypothalamus inflammation during high fat diet induced obesity in mice is sex-specific. Male wild type (WT) (C57BL/6) mice fed high fat diet show increased inflammation in the arcuate region of the hypothalamus with increase microglial activation and recruitment of inflammatory myeloid cells. Male tg21 mice with elevated transgenic EPO production in brain are protected from hypothalamus inflammation associated with high fat diet feeding. Estrogen is protective in female mice against hypothalamus inflammation associated with high fat diet feeding that is only apparent in WT mice after ovariectomy and the protective effects of elevated brain EPO becomes evident in ovariectomized tg21 mice.

Male mice on high fat diet exhibit chronic low-grade hypothalamus inflammation, activation of microglial cells and increased proinflammatory cytokine expression (Valdearcos et al., 2015). On normal chow, *EPOR^(nestinKO)^* mice show minimal difference in hypothalamus inflammation compared with wild-type mice. Endogenous EPO is protective against obesity induced hypothalamus inflammation (Dey et al., 2020). Male *EPOR^(nestinKO)^* mice on high fat diet exhibit greater inflammatory stress, microglial cell activation in the hypothalamus, and recruitment of peripheral myeloid cells compared with wild-type male mice (Table 1). High fat-diet feeding in wild type mice results in increase in metabolic stress with increase in serum ACTH, corticosterone, and C-reactive protein levels. tg21 mice expressing high transgenic EPO in brain are protected from these adverse effects of high fat-diet and show a better physiological control of ACTH, corticosterone, and C-reactive protein (Dey et al., 2020). The male tg21 mice also show lower inflammatory stress markers such as microglial activation, as detected by induction of Iba1 expression, and TNFα secretion in the hypothalamus (Table 1). In comparison to male mice, female mice are protected from high fat-diet induced weight gain and metabolic stress (Dey et al., 2020). In female mice, lack of EPOR in the neuronal cells (*EPOR^(nestinKO)^*) does not worsen the effect of high fat diet and EPO over-expression in the brain (tg21) does not provide any additional protection under such circumstances (Figure 3) (Dey et al., 2020). Estrogen plays a critical role in providing protection and ovariectomy in WT mice abrogates the protective effect against high fat diet induced weight and fat gain and hypothalamus inflammation. In contrast, this metabolic stress and increased hypothalamus inflammation is still prevented in ovariectomized female tg21 mice due to the elevated EPO levels in the brain (Figure 3) (Dey et al., 2020). Female tg21 mice show no difference in serum ACTH, corticosterone, and C-reactive protein during either normal or high fat-diet. Administration of recombinant human EPO in the brain via intracerebroventricular osmotic pump also protects the male mice from gaining weight and fat during high fat diet feeding concomitant with reduced expression of inflammatory markers in the hypothalamus.

### Brain Specific Erythropoietin and Glucose Tolerance

EPO effect in the brain can improve physiological glucose regulation under normal conditions. Under both normal diet and high fat diet, *EPOR^(nestinKO)^* mice show worse glucose tolerance, while the tg21 mice show improved glucose tolerance under similar conditions (Table 1) (Dey et al., 2020). Additionally, this effect is seen in both male and female mice, suggesting that the EPO-mediated control is independent of the protective effect of estrogen. Intracerebroventricular administration of recombinant human EPO can also reduce fasting blood glucose levels. Studies done with the tg21 mouse model suggest that regulation of glucose metabolism is probably carried out by regulation of Fgf21 and adiponectin. Fgf21, a cytokine produced primarily in liver, is involved in regulation of glucose metabolism and insulin sensitivity, and one of the downstream targets of Fgf21 is adiponectin production from white adipose tissue. Increased Fgf21 during diet induced obesity in WT mice was accompanied by lower adiponectin production from white adipose tissue, possibly due to downregulation of the Fgf21 coreceptor βKlotho (Dey et al., 2020). Adiponectin contributes to the metabolic benefits of Fgf21 in both liver and skeletal muscles (Lin et al., 2013). Fgf21 can also cross the blood-brain barrier and act as a messenger between the liver and hypothalamus, by regulating corticotropin releasing factor (CRF) expression and adrenal corticosterone levels (Liang et al., 2014). The release of CRF from the hypothalamus induces secretion of ACTH from the pituitary, that in turn results in the release of corticosterone from the adrenal gland. Although this effect is seen in both male and female mice, the hypothalamus response of Fgf21 with respect to CRF expression showed sexual dimorphism without any difference in Fgf21 receptor and βKlotho expression (Dey et al., 2020). Higher CRF expression in the hypothalamus of male WT mice could ultimately cause higher ACTH and corticosterone levels in the serum.

## Conclusion

EPO response is determined by the extent of EPOR expression and animal models have been useful in demonstrating EPO stimulation of EPOR in ischemic and metabolic stress or injury beyond EPO stimulated erythropoiesis. Metabolic response to endogenous EPO and elevated EPO in the circulation or brain in mice suggest that EPO improves glucose metabolism and provides regulation of fat mass and inflammation associated with diet induced obesity, especially in males. In females, EPO effect in regulation of metabolism is comparable to that in males in the absence of estrogen effects. Relevance of metabolic EPO responses to human is indicated by subset analysis of full-heritage Pima Indians that shows a negative association of endogenous EPO level and percent weight change per year in males and a positive association in females (Reinhardt et al., 2016). The metabolic benefit of elevated EPO and EPO induction with increasing altitude may contribute to the reduced incidence of obesity in military recruits in the United States associated with residence at high altitude (Voss et al., 2014). Animal models provide insight on EPO activity in non-hematopoietic tissue such as bone loss accompanying EPO stimulated erythropoiesis that led to multivariable analysis of USRDS data sets which unexpectedly showed EPO dose as an independent risk factor for hip fracture in hemodialysis patients. EPO treatment in animal models show EPO regulation of glucose homeostasis and obesity associated fat mass and inflammation with contributions from genetic background, sex and expression of EPOR in non-hematopoietic tissue such as fat and brain, and support the potential benefit of EPO in metabolic regulation for human health.
